# Drawing Wellbeing: findings from an art-based exploration with Aboriginal and Torres Strait Islander children

**DOI:** 10.1080/17482631.2026.2644588

**Published:** 2026-03-12

**Authors:** Kate Anderson, Alana Gall, Tasha-Jade Cole, Taleah Carson, Kirsten Howard, Darren Garvey, Michelle Dickson, Martin Howell, Maryanne Theobald, Oliver Black, Justin Wilkey, Cammi Murrup-Stewart, Gail Garvey

**Affiliations:** a Yardhura Walani Centre, Australian National University, Acton, Australia; b First Nations Cancer and Wellbeing Research Program, School of Public Health, The University of Queensland, Brisbane, Australia; c National Centre for Naturopathic Medicine, Faculty of Health, Southern Cross University, East Lismore, Australia; d Leeder Centre for Health Policy, Economics and Data, Faculty of Medicine and Health, University of Sydney, Sydney, Australia; e Curtin School of Population Health, Curtin University, Perth, Australia; f The Poche Centre for Indigenous Health, Faculty of Medicine and Health, University of Sydney, Sydney, Australia; g School of Education, Faculty of Creative Industries, Education and Social Justice, Queensland University of Technology, Brisbane, Australia; h Faculty of Education, University of Melbourne, Melbourne, Australia; i Gukwonderuk Indigenous Health Workforce Centre, Faculty of Medicine Nursing and Health Sciences, Monash University, Clayton, Australia

**Keywords:** Aboriginal and Torres Strait Islander, children, wellbeing, qualitative, Indigenous health, measurement, art-based

## Abstract

**Purpose:**

Aboriginal and Torres Strait Islander children are custodians of the world's oldest living cultures yet experience systemic inequities that infringe upon their rights to health, education, safety, and cultural identity due to ongoing colonisation. Despite these persistent disparities, few studies have explored wellbeing from Aboriginal and Torres Strait Islander children's own perspectives and lived experiences. This study addresses this gap by privileging children's voices in a large-scale, culturally grounded qualitative investigation.

**Method:**

This study represents the first phase of the What Matters 2Kids (WM2K) project, which aims to develop a culturally relevant wellbeing measure for Aboriginal and Torres Strait Islander children aged 5–11 years. Using an Indigenist methodology and culturally responsive Art and Yarning method, 219 Aboriginal and Torres Strait Islander children across 15 sites in urban, regional, and remote Australia participated in creative sessions to express what supports their wellbeing. Data was analysed using Reflexive Thematic Analysis and a Collaborative Yarning approach.

**Results:**

Culture emerged as a foundational element underpinning and connecting all aspects of Aboriginal and Torres Strait Islander children's wellbeing. Within this cultural foundation, seven interconnected themes were identified: *caring relationships, connection to Country and nature, feeling safe, hopes and dreams, strong mind and body, interests and activities,* and *strong identity*. A culturally resonant visual analogy, the “Wellbeing Stones”, was developed to conceptualise these findings.

**Conclusions:**

This study provides critical insights into the holistic, relational, and culturally embedded nature of wellbeing for Aboriginal and Torres Strait Islander children, offering essential groundwork for developing culturally appropriate measurement tools and interventions with important implications for research, policy, and service provision.

## Article highlights

- First large-scale qualitative study privileging voices of Aboriginal and Torres Strait Islander children aged 5−11 years to explore wellbeing from their own perspectives and lived experiences (*n* = 219 children across 15 sites).
- Culture emerged as foundational to wellbeing rather than a separate domain, underpinning and connecting all other aspects through the novel “Wellbeing Stones” conceptual framework.
- Seven interconnected themes identified as central to children's wellbeing: caring relationships, connection to Country and nature, feeling safe, hopes and dreams, strong mind and body, interests and activities, and strong identity.
- Indigenist methodology with Aboriginal and Torres Strait Islander Community Researchers leading all data collection using culturally responsive Art and Yarning methods, ensuring authentic engagement and cultural safety.
- Findings challenge deficit-based models and demonstrate children's holistic, relational understanding of wellbeing, providing essential groundwork for developing culturally appropriate measurement tools.
- Critical policy implications: wellbeing initiatives must be culturally grounded rather than simply adapted, with integrated whole-of-child approaches that recognize interdependencies between identity, relationships, safety, and cultural connection.

## 1. Introduction

Aboriginal and Torres Strait Islander children are the future custodians of the world's oldest living cultures. Despite violent disruptions of colonisation, they continue to carry ancestral, cultural and spiritual connections to the lands and waterways now known as Australia, connections that have persisted, adapted and been reimagined across more than 65,000 years (Clarkson et al., 2017). While these immemorial connections can provide Aboriginal and Torres Strait Islander children with knowledges and networks to support and nurture their wellbeing, deep challenges face this young population due to the ongoing corrosive and persistent processes of colonisation today (AIHW, 2018). Structural inequalities, rooted in dispossession, racism, and ongoing exclusion, contribute to significant disparities in health and wellbeing when compared with non-Indigenous children (Azzopardi et al., 2018). This manifests in diminished mental health, including high rates of suicide, which is the leading cause of death among Indigenous children aged 5−17 years (AIHW, 2018). Addressing these inequities is essential for upholding Aboriginal and Torres Strait Islander children’s rights to health, education, safety, and cultural identity, and requires investing in the strengths, relationships and knowledge systems that have supported their communities across generations (United Nations General Assembly, 2007; United Nations, 1989).

Wellbeing represents a complex construct with multiple competing definitions and conceptualisations (Jarden & Roache, 2023). For this study, we understand wellbeing as a state of health, happiness and contentment, with the parts of life that foster and protect wellbeing being culturally-specific. Ensuring that Aboriginal and Torres Strait Islander children experience wellbeing is essential for their individual development and for the vitality of Australian society. Interactions between children’s cultural strengths and the colonially-created challenges they face are likely to impact the wellbeing of Aboriginal and Torres Strait Islander children differently from adults and adolescents (Jones et al., 2018; Toumbourou et al., 2014). It is therefore critical to identify and understand from the perspective of Aboriginal and Torres Strait Islander children what are the particular factors that comprise and shape their wellbeing.

Dominant Western conceptualisations of wellbeing, which often emphasise individual psychological states and personal achievement (Diener et al., 2018), can be inadequate or even inappropriate when applied to Aboriginal and Torres Strait Islander contexts. Indigenous conceptualisations of wellbeing are inherently relational, encompassing connections to family, community, land, culture, and spirituality in ways that cannot be separated from one another (Garvey et al., 2021). These differences are not merely semantic but reflect fundamentally different worldviews about what constitutes a good life and how wellbeing is experienced, maintained, and transmitted across generations.

Aboriginal and Torres Strait Islander leaders have called for culture to be centred in all efforts to understand and support the health and wellbeing of Aboriginal and Torres Strait Islander Peoples (Backholer et al., 2021). This directive is to ensure that Aboriginal and Torres Strait Islander ways of knowing, being and doing underpin all research, policy and practice that impacts Aboriginal and Torres Strait Islander Peoples. While recognising the linguistic and cultural diversity of Aboriginal and Torres Strait Islander Peoples, it is generally accepted that commonalities exist in understanding and experiencing wellbeing as being multi-dimensional, holistic, and centred around culture, community and Country (Butler et al., 2019; National Aboriginal Health Strategy Working Party, 1989). Emerging research with Aboriginal and Torres Strait Islander children, and Indigenous children globally, suggests that community, culture and Country (also called “land”) are also important foundations for wellbeing for children (AIHW, 2018; Anderson et al., 2022). However, the ways in which these foundations manifest in children’s lives and impact on their wellbeing may differ from other age groups. As children are the experts in their own lived experience and have the right to have a say in matters that affect them, it is crucial that the perspectives of children are included and centred in the collection and synthesis of any evidence about them (United Nations, 1989). To date, little research has focused on understanding the protective factors that support and nurture the wellbeing of Aboriginal and Torres Strait Islander children, with even less that centres on culture (AIHW, 2018; Taylor, 2008).

Accurate, strengths-based data about the wellbeing of Aboriginal and Torres Strait Islander children is needed to inform and evaluate programmes and policies to more effectively support their wellbeing to ensure their rights are met. A strengths-based approach focuses on identifying and building upon the existing capabilities, resources, and positive attributes of individuals and communities, rather than focusing solely on deficits, problems, or needs (Bullen et al., 2023). For Aboriginal and Torres Strait Islander children, this means privileging their cultural knowledge, family connections, and community relationships as sources of wellbeing, resilience, and identity. The currently available standardised wellbeing measures often fail to capture what matters to Aboriginal and Torres Strait Islander children (McLean et al., 2022; OECD, 2021). For example, mainstream wellbeing measures commonly used in Australia, such as the Strengths and Difficulties Questionnaire (Goodman, 1997) and the KIDSCREEN (Ravens-Sieberer et al., 2005), tend to focus on individual psychological functioning and social relationships within Western cultural frameworks. These measures often miss critical dimensions of Aboriginal and Torres Strait Islander wellbeing such as connection to Country, cultural identity, community belonging, and the holistic, relational understanding of health that encompasses spiritual, emotional, and cultural domains (Bourke et al., 2022). Efforts to address existing inequities have been hindered by a dearth of accurate, culturally-responsive wellbeing data (AIHW, 2018).

This paper presents findings from the first phase of the What Matters 2Kids (WM2K) project, funded by National Health and Medical Research Council Ideas Grant (2022/GNT2020636), which is developing a nationally relevant, strengths-based and culturally grounded wellbeing measure for Aboriginal and Torres Strait Islander Australian children aged 5–11 years. The first phase aimed to qualitatively explore and describe the views of Aboriginal and Torres Strait Islander children about the parts of life that comprise child wellbeing. The findings of this study will inform the development of the WM2K measure and provide critical evidence for other researchers, policy makers and service providers to understand what parts of life are important to Aboriginal and Torres Strait Islander children around Australia.

## 2. Methods

### 2.1. Research team

As a research team, we are critically aware of power dynamics inherent in research with Aboriginal and Torres Strait Islander children, particularly given the historical legacy of research being conducted on rather than with Indigenous communities (Smith, 1999). We acknowledge the importance of our backgrounds and perspectives, and reflexively consider how such factors may influence our approach to research (Alvesson & Skoldburg, 2000; Nilson, 2017). Our author team comprised Aboriginal and Torres Strait Islander Peoples (AG, TJC, TC, DG, MD, OB, JW, CMS, GG) and non-Indigenous researchers (KA, KH, MH, MT). Our research roles for this study included: qualitative researchers (KA, AG, DG, MD, OB, GG, MT), Aboriginal and Torres Strait Islander Community Researchers (TJC, TC), health economists (KH, MH), and governance committee members (CMS, JW).

### 2.2. Aboriginal and Torres Strait Islander governance

Aboriginal and Torres Strait Islander leadership and governance throughout the project is a foundational element of Indigenist methodologies and equitable processes. A Co-design Working Group (CWG) was established upon project commencement, consisting of majority Aboriginal and Torres Strait Islander stakeholders and representatives from youth organisations, service providers, schools and community-controlled health services. The CWG met three times during the Art and Yarning study to provide guidance and feedback on the methods, engagement, sites and the data analysis.

### 2.3. Ethics

The research methodology and conduct adhered to the ethical principles outlined in the AIATSIS Code of Ethics for Aboriginal and Torres Strait Islander Research (Australian Institute of Aboriginal & Torres Strait Islander Studies, 2012), ensuring respectful and culturally appropriate engagement with Indigenous knowledge and communities. Ethics approvals were obtained for this phase of the study from: the University of Queensland Human Research Ethics Committee (Ref: 2023/HE000607); Western Australian Aboriginal Health Ethics Committee (Ref: WAHREC1260); Far North Queensland Human Research Ethics Committee (Ref: 2023/QCH/99346); Aboriginal Health and Medical Research Council of New South Wales (Ref: 2140/23); Aboriginal Health Council of South Australia (Ref: 04-23-1063); Menzies School of Health Research and Northern Territory Department of Health Human Research Ethics Committee (Ref: HREC 2023-4608); and the Australian National University Human Research Ethics Committee (Ref: H/2024/0914). Approvals from the Departments of Education were obtained in each state and territory where school sites were engaged in the project. Each team member attending sites attained the relevant jurisdictions’ Working with Children Check.

### 2.4. Indigenist methodology

The current study is described in detail below as per the requirements of the *Consolidated criteria for strengthening reporting of health research involving Indigenous peoples* (the CONSIDER statement) (Huria et al., 2019). The study employed a strengths-based, Indigenist methodology (Rigney, 1999; Smith, 2012) to understand what comprises the wellbeing of Aboriginal and Torres Strait Islander children. Given the enduring process of colonial disempowerment and marginalisation of Aboriginal and Torres Strait Islander Peoples in Australia, the use of Indigenist methodologies and a strengths-based approach was a purposeful decision to avoid further subjugation of this group. We employed an Indigenist research methodology to ensure that Aboriginal and Torres Strait Islander children’s voices guide all aspects of the research. Our research team has extensive experience in using Indigenist research methods to ensure that our work contributes to improving the outcomes and experiences of Aboriginal and Torres Strait Islander Peoples. Our approach was guided by the following principles: ensuring Aboriginal and Torres Strait Islander voices and perspectives are prioritised and privileged throughout all aspects of the research process and our work; building Aboriginal and Torres Strait Islander Peoples’ research capacity and developing future research leaders; and facilitating collaboration through engaging and connecting with a range of key stakeholders.

Throughout the study, we actively negotiated interpretations through ongoing dialogue between non-Indigenous and Aboriginal and Torres Strait Islander researchers, with Community Working Group members, and through returning to children's original data. This iterative process helped ensure that our interpretations privileged Aboriginal and Torres Strait Islander ways of knowing while acknowledging the positionality and potential biases we each brought to the analysis. Joint interpretations emerged through respectful listening, cultural humility, and a commitment to Aboriginal and Torres Strait Islander leadership in determining what the data means.

### 2.5. Art and Yarning method

We adapted the Draw, Write, Tell method (Angell et al., 2014), to ensure that the mode of engagement with children is familiar and empowering, by incorporating an established Indigenist research method called Yarning (Bessarab & Ng’andu, 2010). The subsequent method that incorporates both “Draw, Write, Tell” and “Yarning”, we have termed the *Art and Yarning* method.

Draw, Write, Tell is a creative research technique often used in research involving children. It combines three activities: 1) children are asked to draw pictures related to the research topic; 2) they write about their drawings or the topic; and 3) they verbally explain their drawings and writings (Angell et al., 2014). This method is effective because it allows children to express their thoughts and feelings in multiple ways, accommodating different communication preferences and abilities and respecting cultural knowledge sharing methods. It also ensures the stories and experiences that children share are uniquely their own and are generated without researcher oversight or control, which contributes to the rich story telling outcomes driven by the children. The method has been used in various fields, including health, social care, and education, to gather rich, qualitative data from children (Angell et al., 2014; Noonan et al., 2016).

*Yarning* is an Aboriginal and Torres Strait Islander culturally grounded method of communication, and involves sharing knowledge, listening, interpreting, re-interpreting, and collective sense-making. Yarning in a research context privileges Aboriginal and Torres Strait Islander knowledges by grounding data and data collection in Aboriginal and Torres Strait Islander values and traditions (Bessarab & Ng’andu, 2010). However, it is important to acknowledge that Yarning is not a universal practice across all Aboriginal and Torres Strait Islander communities, and some scholars have challenged its validity when applied outside of appropriate cultural contexts or without proper cultural grounding (Atkinson et al., 2021). In this study, Yarning Circles were chosen as the preferred method specifically because they could be applied in ways that supported the telling of stories and sharing of knowledge between research participants (Atkinson et al., 2021). A Yarning Circle is a traditional Aboriginal and Torres Strait Islander cultural practice where participants sit in a circle to share stories, knowledge, and experiences through respectful dialogue, fostering relationship-building, cultural connection, and collective learning in a safe and egalitarian space (Atkinson et al., 2021). Research Yarning typically comprises of informal social discussion and more formal research-focussed interaction. A culturally significant feature of Yarning is its emphasis on relatedness between researcher and knowledge holders (Noonan et al., 2016). Yarning aims to develop familiarity and trust between parties who may not be known to each other while permitting pre-existing relationships and the knowledge held within these to inform and influence the conduct of the Yarn (Bessarab & Ng’andu, 2010). In the context of this study, attention was paid to encouraging Yarns that not only enabled the participating children to understand the research, but to gain a sense of the researchers involved as people with whom they would like to Yarn with. Pre-existing relationships within the various Art and Yarning groups also meant that questions and comments were more readily posed between children who already knew each other. Crucially, the successful use of Yarning Circles in this research was only possible because Aboriginal and Torres Strait Islander Community Researchers (TJC, TC) led these sessions, bringing the necessary cultural knowledge, relationships, and legitimacy that non-Indigenous researchers could not have provided.

The Art and Yarning method was used to investigate and understand the parts of life that contribute to Aboriginal and Torres Strait Islander child wellbeing. We developed an Art and Yarning protocol to guide these sessions. Aboriginal and Torres Strait Islander Community Researchers (TJC, TC) led the facilitation of all Art and Yarning sessions with children for this study with support from experienced researchers (KA, AG, OB).

### 2.6. Sites and recruitment

Children were engaged from 15 locations across seven Australian states and territories, encompassing a mix of urban, regional, and remote communities (see Table I). Recruitment sites included organisations that support Aboriginal and Torres Strait Islander children, such as schools, youth programmes, sporting clubs, and community groups. These organisations played a vital role in establishing connections between researchers and children, offering a culturally safe and respectful environment where children could choose to participate freely.

**Table I.** Demographics of children in this study.

Demographic measure	*n* (%)
**Gender**	
Male	86 (39%)
Female	133 (61%)
**Age (years)**	
5	20 (9%)
6	26 (12%)
7	23 (11%)
8	40 (18%)
9	31 (14%)
10	37 (17%)
11	42 (19%)
**Indigeneity**	
Aboriginal	180 (82%)
Aboriginal and Torres Strait Islander	25 (11%)
Torres Strait Islander	14 (7%)
**State/Territory**	
New South Wales	58 (26%)
Western Australia	14 (6%)
Queensland	58 (26%)
Northern Territory	9 (4%)
Victoria	25 (11%)
South Australia	31 (14%)
Tasmania	24 (11%)
Remoteness Category*	
Major City	42 (19%)
Inner Regional	8 (4%)
Outer Regional	154 (70%)
Remote and Very Remote	15 (7%)

*Remoteness was classified using postcode and locality data within the Accessibility/Remoteness Index of Australia (ARIA) 2016 (Australian Bureau of Statistics, 2023).

Each site designated a local Liaison responsible for connecting with children who might be interested to participate, securing parental consent, and coordinating logistics for the Art and Yarning sessions. Research team members met with these local liaisons, either in person or virtually, to share details about the project and the planned activities. To support recruitment, local liaisons received information packs explaining the study, and consent documents. These materials were distributed to families ahead of the sessions and completed consent forms were returned to the research team to facilitate planning.

In cases where children expressed interest on the day of the session, staff contacted families to obtain verbal or written consent before including them in the Art and Yarning session. The consent process covered both participation in the study and permission to use photographs of the children’s artwork in future publications and research outputs.

### 2.7. Informed consent

Parents of all children invited to participate received information sheets and consent forms about the study. Written informed parental consent was obtained for every participating child, covering both study participation and permission to use photographs of the children's artwork in future publications and research outputs.

On the day of the Art and Yarning sessions, children with parental consent were engaged in discussion with a facilitator about the study and session to confirm their willingness to participate. Verbal assent was sought from each child before they joined the sessions. Children showing any hesitancy or reluctance could leave at any time without consequence, or alternatively, remain to draw without participating in the Yarning session.

### 2.8. Data collection

Site visits were coordinated between the research team and the local site liaisons. Senior research team members (KA, AG GG) trained and mentored Aboriginal and Torres Strait Islander Community Researchers (TJC, TC) to facilitate the Art and Yarning sessions. This training provided an overview of the research programme and the WM2K project; Art and Yarning methods; overview of their role; ways to deal with children’s distress or safety issues.

Typically, the sessions were run in school or community organisation sites, in a room or outside area with space for children to comfortably sit on the ground or at tables to draw, write and Yarn, depending on their preferences. A group of 8–10 Aboriginal and Torres Strait Islander children were recruited at each site. The local Liaison provided guidance to the research team as to the appropriateness of having gendered or aged-based Art and Yarning sessions depending on site and community preferences.

At each session, food and drink were provided before the session formally began. The Community Researchers then played some games with the children to build rapport and get to know each other. They then explained the purpose of the study to the children and what the Art and Yarning session would entail.

A range of art materials and paper was laid out for children to use. Children were then asked to draw or paint things in their lives that support their wellbeing. For younger children, the meaning of wellbeing was described as when you feel happy and strong. Community Researchers also drew their own pictures while the children were drawing and painting to build rapport and talk with the children while they were drawing and painting. The children were given 15–20 minutes to draw or paint.

When the group had finished their art, the Community Researchers brought the children together in a Yarning Circle to Yarn about their artworks. Sometimes local Liaison joined in the Yarning Circle, but most commonly only TJC and TC ran the Circle with the children. They encouraged children to share their views about the meaning of their drawings and their connection to wellbeing. Each child was invited to share something about their drawing when it was their turn to Yarn with the group and why it was important to them and its connection to their wellbeing. Other children were then invited to Yarn about the topic of the drawing and its connection to wellbeing. Facilitators worked to encourage and support the explanations of the drawings by highlighting elements of the art and reflecting on what the children had said about its meaning. As is expected in Yarning circles, especially with children, each group included children who were more outspoken than others. Facilitation methods were therefore adapted to accommodate this diversity, ensuring that louder and more confident voice were recognised without dominating, while creating space for quieter group members who were less comfortable contributing publicly. Efforts were made to encourage all children to share their thoughts on the art in ways that felt culturally safe and respectful. Where any child did not share in the larger group, a researcher approached them individually to see if they would like to share the meaning of their artwork in a one-on-one format. This ensured that all children had an opportunity to share what made them feel happy and strong, and supports their wellbeing, bringing a diversity of views to all Art and Yarning sessions.

Once all drawings were shared and discussed, children were asked if there was anything else they would like to speak about that supports their wellbeing, or for younger children, what makes them feel happy and healthy. This also provided the facilitator with the opportunity to reflect their initial thoughts back to the group as a means of summarising and checking what they had seen and heard.

The Yarning Circles were audio recorded with permission of children and their parents. The audio recordings were transcribed for analysis. Individual children could not be identified in the audio recordings or the transcripts to ensure that children’s identities remained anonymous and confidential. Transcripts were uploaded into NVivo 12 (QSR International Pty Ltd., 2020) for qualitative data management.

### 2.9. Data analysis

Consistent with an Indigenist research framework, the data analysis process was designed to elevate and prioritise the perspectives of Aboriginal and Torres Strait Islander contributors. This method ensures that Aboriginal and Torres Strait Islander Peoples are actively engaged in all phases of interpreting and understanding the data (Butler et al., 2024). Ethical considerations in involving children in the analysis were paramount. While children were not directly involved in the formal coding and thematic analysis processes, their voices and interpretations remained central through the original data they created and the stories they shared.

Data were analysed using a combination of Reflexive Thematic Analysis and Collaborative Yarning. Braun & Clarke’s Reflexive Thematic Analysis, which is a thematic analysis approach commonly used with Yarning research, recognises and acknowledges the biases that researchers bring to qualitative analysis (Braun & Clarke, 2022). A Collaborative Yarning Methodology was employed, which involves a cyclical and adaptive approach to analysis through ongoing dialogue, reflection, and consensus-building among diverse stakeholders (Shay, 2021).

Analysis was conducted through a series of group sessions involving Aboriginal and Torres Strait Islander researchers, Community Researchers, and members of the CWG. These sessions took place over several weeks, allowing for repeated review and refinement of the findings.

The Collaborative Yarning process distinguished between children's direct contributions (their artwork, writing, and spoken narratives) and adult researchers' and CWG members' interpretations of these contributions. CWG members, who share cultural knowledge and community connections with the children, played a critical role in ensuring that themes and interpretations respected children's intended meanings and cultural contexts (Dockett et al., 2009).

Initial coding was carried out by KA, TJC, and TC to identify patterns and familiarise themselves with the data. These preliminary themes were then further explored and validated through collaborative discussions in the Collaborative Yarning sessions. A final CWG meeting in February 2025 brought together eight members to review and provide feedback on the findings, contributing to the finalisation of the thematic framework.

## 3. Results

### 3.1. Profile of children in this study

A total of 22 Art and Yarning sessions were held across the 15 sites, as multiple sessions were held in some sites to accommodate children’s availability. In total, 219 Aboriginal and Torres Strait Islander children participated in the Art and Yarning sessions (see Table I). Most children identified as female (61%) and were aged between 5–11 years. New South Wales (26%) and Queensland (26%) had the highest number of children, and whilst the site locations were diverse, they did not reflect ABS-defined national distributions (Australian Bureau of Statistics, 2022). Children living in cities (19%) and remote areas (7%) were under-represented compared to national figures (35% and 24% respectively), while those in regional areas were over-represented (74% vs 41%).

### 3.2. Qualitative findings

Our analysis identified seven core components that support and nurture Aboriginal and Torres Strait Islander children’s wellbeing: caring people; Country and nature; feeling safe; dreams and hopes; strong mind and body; interests and activities; and strong identity. The role of culture as the foundation of wellbeing was deeply embedded throughout the children’s reports in the Yarning Circles across each of these seven components. The components are explored below with illustrative quotes from children’s descriptions of their drawings. No identifying information has been provided for the included quotes and drawings to ensure absolute anonymity of the children who participated in this study. The central role of culture in anchoring these components to effectively protect and support children's wellbeing is illustrated below through metaphorical and visual representation. This approach reflects the art-based methods used to gather children's voices in the project and honours Aboriginal and Torres Strait Islander storytelling traditions (Bell, 2020).

#### 3.2.1. Culture as the foundation of wellbeing

**Figure 1.**
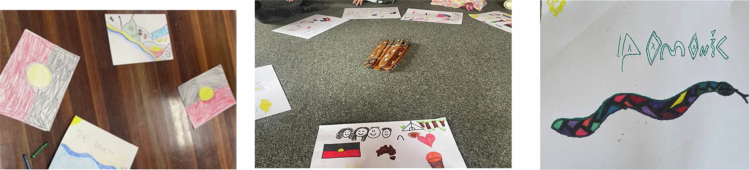
Children’s artwork about their wellbeing.

Across the themes identified in this study, culture emerged as a foundational element that underpins and connects each aspect of wellbeing for Aboriginal and Torres Strait Islander children (see Figure 1). As represented in the following quotes from children, descriptions of culture were woven throughout their drawings and descriptions of wellbeing.

*“We dance about our culture, country and animals.”*

*“I drew a fire with people around it having a Yarn.”*

*“I respect my ancestors and what they use to do for us.”*

*“I feel connected to nature when doing art.”*

Children’s expressions of wellbeing were not isolated experiences but deeply embedded in cultural knowledge, practices, relationships, and places. Whether describing the joy of dancing with family, the calm of being on Country, or the pride in seeing the Aboriginal and Torres Strait Islander flags, children consistently linked their wellbeing to cultural identity. Culture was not just one theme among others, it was the grounding force that gave meaning, stability, and strength to all other aspects of wellbeing.

Ethical considerations in involving children in the analysis were paramount. While children were not directly involved in the formal coding and thematic analysis processes, their voices and interpretations remained central through the original data they created and the stories they shared. The Collaborative Yarning process distinguished between children's direct contributions (their artwork, writing, and spoken narratives) and adult researchers' and Community Working Group members' interpretations of these contributions. To represent this interconnected and culturally grounded understanding of wellbeing, members of our team (KA, TJC, TC) engaged in Collaborative Yarning to develop a visual and conceptual analogy called “Wellbeing Stones”.

Within this conceptualisation, Aboriginal and Torres Strait Islander child wellbeing is represented as a small pool in a river, protected by stones that are grounded on a riverbed. The riverbed represents culture. It is stable, supportive, and the path through which life flows. It incorporates knowledge, Country, and ancestors. Culture is the foundation that holds everything together. The stones represent the parts of life that support a child’s wellbeing, the seven themes identified in this study. Therefore, an Indigenous child’s wellbeing requires that these stones must be strong and structured together in a way that protects and supports the pool in the middle, their wellbeing.

The river represents the child’s experience of life, as it flows around and through the stones, sometimes smooth, sometimes rough. As the river flows through and around the pool, the strength and connection of the stones, and their grounding in the riverbed, help to keep the pool calm and stable. If the stones shift, get damaged, or lose their connection to the riverbed, the water in the pool can become disturbed. This disruption can weaken the protective positioning of the stones, making children more vulnerable to being impacted by life’s challenges.

This analogy illustrates how the parts of life that are important to an Aboriginal and Torres Strait Islander child must be in place and culturally grounded to keep their wellbeing calm and stable. If these supports are disconnected from culture or not positioned well to protect the child, then their wellbeing is more vulnerable to disturbance. Collaborative Yarns identified that checking on the strength and cultural grounding of the “stones” in a child’s life can offer insight into how their wellbeing is faring amidst life’s ups and downs.

#### 3.2.2. Caring people

The drawings and voices of children in this study communicate the central role that caring relationships play in their wellbeing. Children described how family, friends, and community members contribute to their sense of safety, happiness, and belonging. Children consistently emphasised the joy and comfort they experience when spending time with family. These moments, whether routine or celebratory, were described as very important to their wellbeing. The data revealed that caring relationships, whether with family, friends, or through cultural connections, are deeply intertwined with Aboriginal and Torres Strait Islander children’s understanding of wellbeing. These relationships provided emotional support, safety, joy, and a sense of identity. The children affirmed that wellbeing is not an individual experience but one rooted in connection, care, and community.

The importance of shared experiences and the creation of lasting memories with loved ones was a recurring theme.

*“I like having sleepovers with my cousins because I love them. And I love playing with them.”*

*“Roasting marshmallows with family”*

*“Making breakfast for my family”*

*“Footy game that my family went to”*

*“The park because that’s where my family meets up and has family day”*

Safety was a common theme amongst children who expressed how being near parents and carers made them feel secure and cared for. These expressions highlighted the emotional and physical protection that children associate with their caregivers, reinforcing the foundational role of family in their wellbeing.

*“I love my mum because she protects me and helps me when I get hurt.”*

*“I love my dad because whenever I am near him, I know there is nothing to be scared of. He helps me and won’t let anybody hurt me.”*

*“My nan because she always makes a soup for me when I am sick”*

*“My parents protect me and help when I am hurt or scared”*

Children also spoke about places that hold strong family connections, describing them as sources of comfort and identity. These places were not just geographic locations but are imbued with relational and cultural significance, contributing to a sense of belonging and wellbeing.

*“I like going[location], my families live there. It’s home.”*

*“Family time in[location]”*

*“My hometown –[location]—spending time there with friends and cousins”*

Friendship was another key aspect of wellbeing, particularly in the school context. Children described friends as sources of happiness, support, and emotional resilience. The Yarns showed that friendships were not only social connections but also emotional anchors that helped children navigate challenges and celebrate joys.

*“Friends are always there when I am upset.”*

*“Friends and family make my life better and make me happy.”*

*“Friends are family at school.”*

#### 3.2.3. Country and nature

Children in this study expressed a deep and meaningful connection to Country, describing it as a source of peace, joy, and cultural identity. Their reflections highlighted how being outdoors, engaging with animals, and experiencing natural environments contributed significantly to their wellbeing.

Children described nature as a place where they feel calm, relaxed, and emotionally restored. The sensory experiences of being outdoors, such as fresh air, natural sounds, and sunlight, were frequently mentioned as soothing and enjoyable.

*“Nature gives you air, it is beautiful and calm where you can relax.”*

*“Listen to the sounds of nature”*

*“The ocean is nice and peaceful”*

*“A rainbow out the window because it is colourful and beautiful”*

*“I love the sun - it keeps you warm.”*

These quotes suggest a therapeutic quality of Country and its role in supporting children’s emotional wellbeing.

Children spoke about the importance of Country not just as a physical space, but as a cultural and spiritual anchor. Being “on Country” and engaging with cultural stories and animals helped them feel connected to their ancestry and community.

*“Country is important to me.”*

*“Swimming on Country”*

*“Listening to cultural stories and knowledge”*

*“Emu and Kangaroo footprints”*

These reflections build on the notion that Country is therapeutic, illustrating how Country serves as a living connection to culture, family, and identity.

Children also communicated their love for animals and outdoor play, describing these experiences as joyful and central to their daily lives.

*“I like animals, flowers and nature—being outside.”*

*“I respect our Country and the animals.”*

*“I like coming home and playing with my puppy.”*

*“Watering mum’s garden—it makes me feel great”*

*“Swimming in creek”*

*“Dancing in the rain”*

Children described wellbeing as being nurtured through meaningful relationships with people, Country, animals, nature, and culture. They indicated that these environments provided opportunities for play and exploration while reinforcing their cultural identity.

#### 3.2.4. Feeling safe

Feeling safe appeared to be a foundational aspect of wellbeing for children in this study. Children described safety not only in terms of physical protection but also emotional comfort, often linked to specific places and relationships. The theme of feeling safe was deeply intertwined with place, relationships, and routine in the lives of children. Homes, schools, and community spaces, especially those associated with loved ones, served as anchors of emotional security.

Children consistently identified their homes as places of safety and calm. The presence of loved ones and the physical structure of the house itself were described as protective and reassuring.

*“My house because it makes me feel safe”*

*“Home—being in a house with a roof over head”*

*“House because it protects the people who live there”*

Children described stable, secure housing environments as important for their emotional wellbeing. They also described specific locations associated with important people, such as their mum’s or nan’s house, as safe spaces that contributed to their wellbeing. Children characterised their homes or homes of loved ones as more than physical shelters, describing them as spaces that provided emotional safety through supportive relationships.

*“Where my mum lives. That’s my safe place; on school holidays I get to go there.”*

Some children identified school as a place where they felt safe and enjoyed spending time. These children attributed these positive feelings to the presence of friends, structured routines, and opportunities for play. Children described schools positively when they perceived them as safe, inclusive, and supportive environments.

#### 3.2.5. Dreams and hopes

Children shared their hopes and dreams with enthusiasm and pride, revealing how aspirations and challenges contribute positively to their wellbeing. These reflections showed that looking forward to the future, setting goals, and imagining possibilities were deeply meaningful experiences that fostered motivation, joy, and self-worth. Aspirations, whether personal, educational, or family-oriented, seemed to be sources of pride and motivation for the children, as many spoke with enthusiasm about things that they wanted to achieve. The opportunity to dream, learn, and grow is central to their understanding of wellbeing, reinforcing the value of nurturing environments that support goal setting and exploration.

Children expressed excitement about upcoming events and transitions, such as birthdays and moving into new school grades. These milestones were associated with growth, learning, and new challenges.

*“Going into a new grade and getting new challenges”*

Children shared a wide range of dreams for their future lives, including lifestyle aspirations, travel, and career goals. These dreams reflected both individual interests and family hopes.

*“My dream is moving to[location].”*

*“Travel to snow with mum”*

*“I love travelling and going to different places. My favourite place is [location].”*

*“My family want to win the lotto and buy a big new house to have a cat and dog.”*

During Yarning Circles, when sharing the things that they liked doing, some children described moments of achievement and the pride they felt when working toward and reaching their goals. Whether in sports, learning, or personal growth, these experiences were seen as affirming and empowering.

*“Winning generally”*

*“Playing rep footy”*

*“Learning and playing on my iPad”*

*“Learning about science and what is good for my wellbeing”*

*“Going to school to learn and do lots of things.”*

Children described experiences of challenge and accomplishment as important for building their confidence and wellbeing.

#### 3.2.6. Strong mind and body

Children described wellbeing as a balance of emotional, physical, and relational experiences. Their reflections on feeling healthy, calm, and happy reveal how everyday activities, especially those involving creativity, food, and caring for others, contributed to a strong sense of wellbeing. They highlighted the importance of everyday practices, drawing, cooking, eating, and relaxing, as essential to feeling healthy, calm, and happy.

Acts of care, especially preparing food for siblings, were frequently mentioned as sources of happiness and pride. These moments reflected how many of the students included nurturing relationships and responsibilities as contributing to a strong sense of self and emotional wellbeing.

*“I always make breakfast for my baby sister and my big sisters. It makes me feel happy.”*

*“I love making breakfast for my family.”*

*“I love cooking for my sister - making pancakes and noodles.”*

Children appeared to derive joy and meaning from caring for others, reinforcing a relational nature of wellbeing.

Children also described food collection, preparation and eating as sources of nourishment, pleasure and cultural connection. Their reflections about the importance of food to their wellbeing included descriptions of their favourite foods and traditional meals.

*“My fav food is potatoes.”*

*“Cooked kangaroo stew”*

*“Eating turtle and crocodile”*

These quotes indicate that importance of food in providing children sustenance, comfort, and joy, as well as supporting their connections with culture.

Cooking was described not only as a fun activity but also to care for others and feel proud. Children shared their enjoyment of preparing meals and helping around the house.

*“I love cooking mac and cheese, pancakes and noodles. I cook kangaroo stew.”*

*“Cooking is more fun to do than to eat.”*

*“Helping at home—doing the dishes”*

These quotes highlight how food-related activities can foster a sense of responsibility, pride, and connection for children.

Children acknowledged the importance of rest and relaxation as part of feeling well, showing an intuitive understanding of balance in daily life.

*“I love sleeping and being lazy.”*

#### 3.2.7. Interests and activities

Children in this study described a wide range of interests and activities that contribute to their wellbeing. These hobbies and pursuits, whether creative, physical, or social, were associated with happiness, pride, and personal growth. Engaging in meaningful activities helped children learn new skills, express themselves, and feel connected to others and to nature.

Children frequently mentioned art, drawing, craft and music as activities that bring joy and help them feel calm. These creative practices were described as peaceful, fun, and emotionally grounding, helping them manage emotions and self-regulate.

*“I love art so there is a paint colour.”*

*“Drawing and painting makes me happy and laugh, it’s fun and peaceful to do.”*

*“Listening to music”*

*“Playing music—my family is very musical”*

*“I like colouring in and drawing. I feel good. And when I am angry it makes me calm.”*

These reflections show how creative activities support emotional wellbeing and offer children a way to express themselves and manage feelings.

Children expressed enthusiasm for outdoor play, sports, and adventure. These activities were associated with fun, friendship, and physical wellbeing.

*“Fishing with pop makes me feel happy.”*

*“Riding motorbikes and going out bush. I Feel happy.”*

*“Sports keeps you active and you get to see friends.”*

*“Cartwheels on the grass at home”*

*“Beach, boats, swimming.”*

Children also described enjoyment in using technology and engaging with media, such as gaming, music, and television. These activities were seen as fun and relaxing.

*“Gaming. I love playing on Xbox, I love the games on it (Hot Wheels).”*

*“My Xbox because I love it and love the games on it.”*

*“Watching and reading anime”*

Children expressed pride in their hobbies and a willingness to keep trying even when things are hard. This theme of persistence was linked to personal growth and self-esteem. Children’s reflections affirmed that engaging in interests and activities helped children build resilience and confidence.

*“I do things that make me feel proud.”*

*“I keep trying even when I fail.”*

#### 3.2.8. Strong identity

Children in this study expressed a deep sense of pride in their cultural identity. Their reflections in the Yarning circles revealed that knowing who they are, participating in cultural practices, and connecting with symbols of identity, such as the Aboriginal and Torres Strait Islander flags, were powerful contributors to their wellbeing. These expressions of identity fostered belonging, strength, and self-love. In the Yarns, children affirmed that knowing who they are, and being proud of it, gave them strength, joy, and a sense of belonging.

*“I drew the Aboriginal flag, being Aboriginal makes me feel good.”*

*“The Aboriginal flag because it means I am an Aboriginal person”*

*“I love being Aboriginal—I love to join in a lot of Aboriginal things.”*

*“I love me” and drew Aboriginal flag with “be kind to others.”*

*“Torres Strait Islander flag”*

Children described participating in cultural activities, such as dancing, storytelling, and art, as experiences that made them feel strong, connected, and joyful. These activities were often shared with family and community, reinforcing intergenerational bonds.

*“I love dancing with Aboriginal dancing. Because I get to dance with my cousins and uncle.”*

*“Learning new cultural things, and the food.”*

*“I love being Aboriginal and learning language and the meaning behind Aboriginal culture.”*

Children expressed reverence for their ancestors and a strong connection to Country, highlighting the spiritual and cultural dimensions of identity.

*“I respect our Country and the animals.”*

*“That’s a Yarning circle.”*

*“My brother collected an emu egg.”*

## 4. Discussion

To our knowledge, this is one of the first large-scale, culturally grounded qualitative studies to explore the wellbeing of Aboriginal and Torres Strait Islander children aged 5–11 years. Through a co-designed, art-based and culturally informed approach, this study captured children’s voices and privileged their perspectives on what supports their wellbeing and what contributes to a good life for them. Children demonstrated a clear awareness of the things that help them feel strong, safe, and happy, and articulated aspirations for their futures. Their reflections revealed a deeply relational and culturally embedded understanding of wellbeing. Our analysis identified seven interconnected themes that describe the parts of life that support wellbeing: *caring relationships, connection to Country and nature, feeling safe, hopes and dreams, strong mind and body, interests and activities, and strong identity*. Importantly, these themes were not experienced in isolation. Rather, they were described as overlapping and mutually reinforcing, with culture emerging as the foundation that underpins and connects them all.

### 4.1. Implications for research, service provision, and policy

#### 4.1.1. Culture as the foundation of wellbeing

This study reinforces that culture is not a separate domain of wellbeing but the foundation upon which all other aspects rest and are shaped. Aboriginal and Torres Strait Islander children’s wellbeing is deeply embedded in cultural identity, connection to Country, and relationships with family and community. While a number of existing models and frameworks include culture as a domain of wellbeing for Aboriginal and Torres Strait Islander Peoples (Anderson et al., 2025; Department of the Prime Minister & Cabinet, 2017; Garvey et al., 2021, 2024), our findings suggest that culture is the underlying foundation that connects and supports the domains of wellbeing. Our findings support the premise that culture provides the values, knowledge systems, and relational structures that shape how wellbeing is understood and experienced. For policy and practice, this means that wellbeing initiatives must be culturally grounded and aligned, not simply culturally adapted, and must reflect Aboriginal and Torres Strait Islander worldviews and values. Programmes that fail to embed culture risk overlooking the very foundations of wellbeing for Aboriginal and Torres Strait Islander children.

By centralising culture as the foundation of Aboriginal and Torres Strait Islander children's wellbeing, this study contributes to conceptualisations of cultural wellbeing (Dockery, 2020), which extends beyond existing relational wellbeing frameworks (Atkinson et al., 2020), by explicitly positioning culture not as one domain among many, but as the underpinning foundation that gives meaning and context to all relational connections. This distinction is critical for understanding how wellbeing is experienced and sustained in Aboriginal and Torres Strait Islander contexts.

#### 4.1.2. Holistic and interconnected wellbeing

The seven themes identified in this study were not experienced in isolation but as interconnected parts of a whole. This reflects the holistic perspective of social and emotional wellbeing described in national frameworks, where wellbeing is understood as the interplay between physical, emotional, cultural, and spiritual dimensions (Anderson et al., 2025, 2022; Garvey et al., 2021). The wellbeing stones analogy developed in this study illustrates how protective factors—such as relationships, identity, and cultural connection—must be strong and grounded in culture to buffer Aboriginal and Torres Strait Islander children from life’s challenges. This has implications for early intervention and prevention strategies, which should focus on strengthening these “stones” in children’s lives. Programmes that support cultural identity, family connection, and safe environments are critical to maintaining wellbeing through life’s ups and downs. For example, initiatives that promote cultural mentoring, intergenerational storytelling, and community-led education can reinforce protective factors and build resilience. For service delivery, this challenges fragmented models and supports integrated, whole-of-child approaches that respond to the full spectrum of children’s needs. For example, a child’s sense of safety at school may be closely tied to their cultural identity and relationships with peers and staff. Recognising these interdependencies is essential for designing effective, culturally safe interventions.

#### 4.1.3. Wellbeing measurement

Available wellbeing measures are predominantly developed for non-Aboriginal and Torres Strait Islander peoples and fail to capture what matters to Aboriginal and Torres Strait Islander children (McLean et al., 2022; OECD, 2021). This study provides foundational evidence for the development of culturally relevant wellbeing indicators, grounded in children’s lived experiences and cultural contexts. These indicators should reflect strengths-based, relational, and culturally specific understandings of wellbeing, as advocated in national policy reforms (Department of the Prime Minister & Cabinet, 2017). Measurement tools must be co-designed with communities and children, and should incorporate visual, narrative, and participatory elements (McLean et al., 2022; OECD, 2021). Importantly, these tools should be used not only for assessment but also to guide reflection, dialogue, and support planning with children and families.

We acknowledge that translating the rich, relational understanding of wellbeing expressed in this qualitative work into quantitative assessments involves inherent limitations. Numerical scoring systems risk reducing the holistic, interconnected nature of wellbeing into discrete components. However, when developed through co-design with Aboriginal and Torres Strait Islander communities and used as tools for dialogue rather than definitive assessment, quantitative measures can complement qualitative approaches (Williamson et al., 2010). The key is ensuring that any quantitative tool serves as a starting point for conversation, supporting culturally grounded participatory practices where families and children can elaborate on and contextualise their responses through storytelling and reflection.

#### 4.1.4. Reframing wellbeing

This study contributes to the growing body of work that reframes wellbeing through Indigenous knowledge systems, moving away from deficit-based models and affirming the right of Aboriginal and Torres Strait Islander Peoples to define and assess their own wellbeing (United Nations General Assembly, 2007; United Nations, 1989). This study further hones this frame by ensuring that Aboriginal and Torres Strait Islander children are guiding the narrative around what is important to their wellbeing. This reframing is essential for developing policies, practices, and measures that are not only effective but also respectful and empowering of children’s rights. This approach also challenges dominant paradigms that position wellbeing as an individualised, clinical construct, instead recognising it as relational, collective, and culturally embedded (Gall et al., 2025). Future research and policy must continue to centre Indigenous voices and knowledge in the design, implementation, and evaluation of wellbeing initiatives (Ngampromwongse & Gall, 2025).

### 4.2. Strengths and limitations

This research faced certain limitations, notably the disproportionately higher number of girls and children from outer regional locations in the sample. Such an imbalance may have influenced the prominence and framing of specific themes, potentially reflecting gender- or location-based differences in perceptions of wellbeing. Furthermore, the relatively few children from very remote communities presents another gap in representation. As a result, perspectives unique to these populations may not have been captured during data collection. Future studies should aim to address these recruitment limitations to ensure the findings are inclusive of children living in very remote areas. Despite these limitations, this study provides a methodologically and culturally robust overview of the parts of life that are important to, and support, the wellbeing of Aboriginal and Torres Strait Islander children nationally. Our methods were underpinned by Indigenist research principles, and the research engagement with children throughout the sessions was conducted with developmental and cultural sensitivity and expertise by our Community Researchers. The findings of this study offer a unique nationally-relevant dataset to guide the development of strength-based, culturally responsive wellbeing measures for Aboriginal and Torres Strait Islander children.

### 4.3. Conclusions

This study offers a significant and culturally grounded contribution to understanding the wellbeing of Aboriginal and Torres Strait Islander children aged 5–11 years. By centring children’s voices through creative and culturally responsive methods, it reveals a holistic and relational view of wellbeing deeply grounded in culture. The seven interconnected themes identified, anchored by the foundational role of culture, highlight the variety and richness of children’s lived experiences. The river and stones analogy developed through this research provides a powerful and culturally resonant framework for conceptualising Aboriginal and Torres Strait Islander children’s wellbeing, with implications for research, policy, and practice. Moving forward, it is essential that wellbeing initiatives are co-designed with communities, reflect Aboriginal and Torres Strait Islander knowledge systems, and prioritise strengths-based, child-centred approaches. This reframing not only enhances the relevance and effectiveness of wellbeing strategies but also affirms the rights of Aboriginal and Torres Strait Islander children to define and shape their own pathways to a good life.

## Data Availability

Data may be available from authors on request.
